# Exploring the Possible Mechanism and Drug Targets of Huang-Qi-Gui-Zhi-Wu-Wu Decoction for the Treatment of Chemotherapy-Induced Peripheral Neuropathy on Network Pharmacology

**DOI:** 10.1155/2020/2363262

**Published:** 2020-11-22

**Authors:** Jia-lin Gu, Guo-li Wei, Yu-zhu Ma, Jin-zhi Zhang, Yi Ji, Ling-chang Li, Jia-lin Yu, Can-hong Hu, Jie-ge Huo

**Affiliations:** ^1^Department of Oncology, Affiliated Hospital of Integrated Traditional Chinese and Western Medicine, Nanjing University of Chinese Medicine, Nanjing, Jiangsu 210028, China; ^2^Graduate School, Nanjing University of Chinese Medicine, Nanjing, Jiangsu 210046, China; ^3^Department of Oncology, Jiangsu Province Academy of Traditional Chinese Medicine, Nanjing, Jiangsu 210028, China

## Abstract

Chemotherapy-induced peripheral neuropathy (CIPN) is a common side effect of anticancer treatment, which may influence its successful completion. The Huang-Qi-Gui-Zhi-Wu-Wu decoction (HQGZWWD) has been widely used to treat CIPN in China although the pharmacological mechanisms involved have not been clarified. Using the network pharmacology approach, this study investigated the potential pathogenesis of CIPN and the therapeutic mechanisms exerted by the HQGZWWD herbal formula in CIPN. The targets of HQGZWWD were identified using traditional Chinese medicine (TCM) databases (TCMSP and ETCM) and prediction platforms (PharmMapper and TargetNet), and the genes of CIPN were collected by DisGeNET, GeneCards, and literature search. The common target interaction network between herbal formula and diseases was constructed by using Cytoscape. Gene Ontology (GO) function and Kyoto Encyclopedia of Genes and Genomes (KEGG) pathway enrichment analysis were used to reveal the mechanism and efficacy of HQGZWWD in the treatment of CIPN. A total of 153 CIPN-related genes were screened, and a protein-protein interaction (PPI) network with 96 nodes and 424 edges was constructed. Sixty-three active components were retrieved from HQGZWWD, with a herb-composite compound-target network including 748 nodes and 5448 edges. Forty-one targets belong to the above two networks. The analysis of network results and literature review shows that the main pathological processes of CIPN may be the inflammatory response and nerve injury, and HQGZWWD plays a therapeutic role in CIPN by regulating inflammatory response and repairing nerve injury, thus verifying the reliable efficacy of this herbal formula. In addition, we found two new potential therapeutic targets (CDK7 and GSTM2) warranting further investigation. This study fully illustrates that TCM has the characteristics of a multicompound, multitarget, and multipathway treatment, which is of great significance to study the curative effect of herbal formulations.

## 1. Introduction

Chemotherapy-induced peripheral neuropathy (CIPN) is one of the most common side effects of chemotherapy with platinum drugs, taxanes, *Catharanthus roseus* alkaloids, thalidomide, and bortezomib. It has been reported that the total incidence of CIPN is more than 60%, while for oxaliplatin and taxanes, rates may be as high as 90% [1]. The occurrence of CIPN is dose dependent and may be relieved by dose reduction or drug withdrawal. The emergence of CIPN seriously affects the efficacy of chemotherapy and the quality of life of patients [2]. CIPN usually occurs in the early stages of chemotherapy; its typical clinical manifestations include sensations of symmetrical burning or tingling, loss of sensation, and numbness at the ends of the extremities. Physical examination reveals fine motor impairment and sensory ataxia, as well as motor neuropathy and proprioceptive impairment, such as myasthenia and muscle bundle tremor [3, 4]. Further, autonomic neuropathy such as abdominal pain, diarrhea, constipation, postural hypotension, and laryngospasm are often accompanying symptoms. Neurogenic pain is also a clinical symptom, which is more likely to have more of a physical and mental impact on patients, and often needs auxiliary pain relief and antidepressant therapy [5]. The emergence of CIPN will lead to tension in patients, affect their quality of life, reduce the treatment dose, prolong chemotherapy treatment, or even stop treatment, thus interfering with the achievement of the desired effect.

The management of CIPN has entered a stage of comprehensive treatment. Traditional Chinese Medicine (TCM), as an important part of the Complementary and Alternative Medicine (CAM) approach, plays a key role. Increasing data show that TCM has an obvious efficacy in the prevention and treatment of cancer and its complications, including radiotherapy, chemotherapy, and postoperative recovery [6–8].

The Huang-Qi-Gui-Zhi-Wu-Wu decoction (HQGZWWD) was first published in the *Synopsis of Golden Chamber* written by Zhang Zhongjing, a medical scientist in the Han Dynasty (25 AD), and represents one of the more commonly used clinical herbal formulations. It consists of five herbs: *Hedysarum Multijugum Maxim* (Huang Qi), *Cinnamomi Ramulus* (Gui Zhi), *Paeoniae Radix Alba* (Bai Shao), *Zingiber Officinale Roscoe* (Sheng Jiang), and *Jujubae Fructus* (Da Zao). Its main function is to supplement qi, the warming meridian, and to ease pain. HQGZWWD is mainly used for the hand-foot syndrome, CIPN, diabetic peripheral neuropathy, and rheumatoid arthritis. Previously, we have confirmed the efficacy of HQGZWWD in the prevention and treatment of CIPN without reducing antitumor activity of chemotherapeutic drugs in the animal model and cancer patients [9]. However, the potential therapeutic mechanism involved has not been fully elucidated.

Network pharmacology is based on the similarity of the structure and efficacy of drugs to construct a drug-target network to explore the mechanisms of action of different agents. It emphasizes the multichannel regulation of signaling pathways to improve the therapeutic effects of drugs and to reduce their toxicity and adverse effects, so as to improve the success rate of new drug clinical trials and save on the costs involved in drug research. Network pharmacology is a current research hotspot applied to clarify the effective components and treatment activity of TCM [10]. It has been widely used to explore the therapeutic mechanisms of TCM in diseases involving the cardiovascular and nervous systems, respiratory diseases, diabetes, cancer, and osteoarthropathy and has achieved significant results [11–16]. The purpose of this study was to analyze the pathogenesis of CIPN and the potential therapeutic mechanisms of HQGZWWD through network pharmacology, so as to provide a theoretical basis for the clinical application of herbal formulations.

## 2. Methods of Data Preparation

### 2.1. Composite Compounds of HQGZWWD

ADME refers to the process of absorption, distribution, metabolism, and excretion of exogenous compounds, which can reflect the dynamic changes of drug activity in animals or humans. It is an important guide to the development of new drugs and to the design of compounds [17]. We searched the Traditional Chinese Medicine Systems Pharmacology Database and Analysis Platform (TCMSP, http://tcmspw.com/tcmsp.php), which is the most widely used information query platform for TCM, to identify the composite compounds and targets of HQGZWWD [18]. A total of 790 compounds were identified: 87 in *Hedysarum Multijugum Maxim*, 220 in *Cinnamomi Ramulus*, 85 in *Paeoniae Radix Alba*, 265 in *Zingiber Officinale Roscoe*, and 133 in *Jujubae Fructus*. All molecules were screened according to the ADME criteria (oral bioavailability ＞30% and drug likeness ＞0.18) recommended by TCMSP [19, 20]. A total of 63 compounds were selected. Notably, 14 of these effective compounds were verified by ultraperformance liquid chromatography-quadrupole-time-of-flight mass spectrometry (UPLC-Q-TOF-MS) technology in our previous study, and several compounds are included: *jaranol*, *isorhamnetin*, *formononetin*, *calycosin*, *kaempferol*, (*3R*)*-3-*(*2-hydroxy-3,4-dimethoxyphenyl*)*chroman-7-ol*, *quercetin*, *sitosterol*, (*+*)*-catechin*, paeoniflorin, benzoyl paeoniflorin, and poriferast-5-en-3beta-ol (Supplementary Tables S1 and S2).

### 2.2. Compound Targets of HQGZWWD

In order to make the targets of HQGZWWD more comprehensive, we have added descriptions of target information from the Encyclopedia of Traditional Chinese Medicine (ETCM, http://www.nrc.ac.cn:9090/ETCM/). The ETCM was updated in 2018 and provides a more professional ADME evaluation and enhanced information on compounds and targets [21]. Based on the limitations of the database, we also used PharmMapper (http://lilab-ecust.cn/pharmmapper/) and TargetNet (http://targetnet.scbdd.com/), both of which are target prediction platforms. The former predicts the target using a pharmacophore model, which models the molecular docking of compounds through characteristic features and the spatial arrangement of pharmacologically active molecules [22]. TargetNet builds a large number of quantitative structure activity relationship (QSAR) models based on the input compounds to predict targets [23]. Next, UniProt (https://www.uniprot.org/) was used to standardize all target names. Some compounds for which the target information could not be identified were excluded. In total, 48 compounds were selected (Supplementary Table S3).

### 2.3. Targets of CIPN

#### 2.3.1. Targets from Known Databases

The molecular targets for CIPN were obtained from GeneCard (https://www.genecards.org/) and DisGeNET (https://www.disgenet.org/). GeneCard is an authoritative platform for human gene annotation information that integrates human gene information from other databases [24]. DisGeNET integrates a large number of data about disease-associated genes and variants from multiple sources, covering almost all areas of human diseases [25]. In addition, we searched the PubMed database (https://www.ncbi.nlm.nih.gov/pubmed/) to supplement information on the disease gene.

#### 2.3.2. Target Supplement

GeneMANIA (http://genemania.org/), developed by the University of Toronto, was used to analyze and identify dominant genes and provided genomic and proteomic data to discover functionally similar genes [26]. We uploaded the disease targets downloaded from GeneCard and DisGeNET to the database and output a list of 20 resultant genes.

Through database screening, literature search, and prediction target supplements, a total of 153 disease genes were selected (Supplementary Table S4).

### 2.4. Protein-Protein Interaction Network

The protein-protein interaction (PPI) networks in this article were all derived from String V11.0 (https://string-db.org/), which was updated in November 2018, to search for functional interactions between proteins and help to mine core regulatory genes in the network [27].

### 2.5. Network Construction

We constructed three networks: (i) the CIPN network, (ii) the herbal-compounds-targets network for HQGZWWD, and (iii) the HQGZWWD-CIPN overlapping targets network. All networks were built through the network visualization software Cytoscape (V3.7.2 https://cytoscape.org/), an open source network that focuses on data visualization and analysis. Its core function is to provide a basic functional visual layout and query network based on the combination of basic data [28]. In addition, we modularized the CIPN network through the Mcode cluster function in Cytoscape, which is conducive to better mining the core functions of the network.

### 2.6. Gene Ontology Functional and Kyoto Encyclopedia of Genes and Genomes Pathway Enrichment Analysis

The Gene Ontology (GO) function and Kyoto Encyclopedia of Genes and Genomes (KEGG) pathway enrichment analyses of all targets were obtained from the Database for Annotation, Visualization and Integrated Discovery (DAVID, V6.8, https://david.ncifcrf.gov/), which integrates biological data and analysis tools to provide comprehensive biological function annotation information for the large-scale gene or protein lists [29].

## 3. Results

### 3.1. The CIPN Network

**Figure 1:**
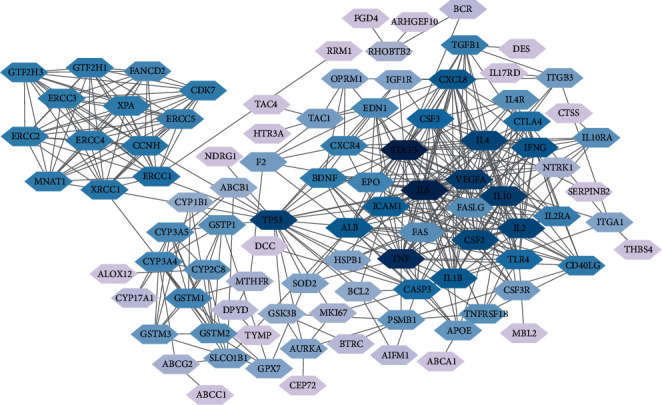
CIPN target protein-protein interaction network. Nodes with a darker color have a higher degree in the network.

Information relative to 150 targets was extracted from String, and 96 nodes and 424 edges were constructed under the condition of high confidence. In this network, targets with a higher degree are regarded as the core targets (32 in STAT3, 31 in IL6, 28 in TNF, 24 in IL10, 24 in VEGFA, 23 in TP53, 22 in IL2, 22 in IL4, and 21 in CSF2). This suggests that these genes may be the key genes or central genes affecting the development of CIPN (Figure 1).

### 3.2. GO Functional Analysis of the CIPN Network

**Figure 2:**
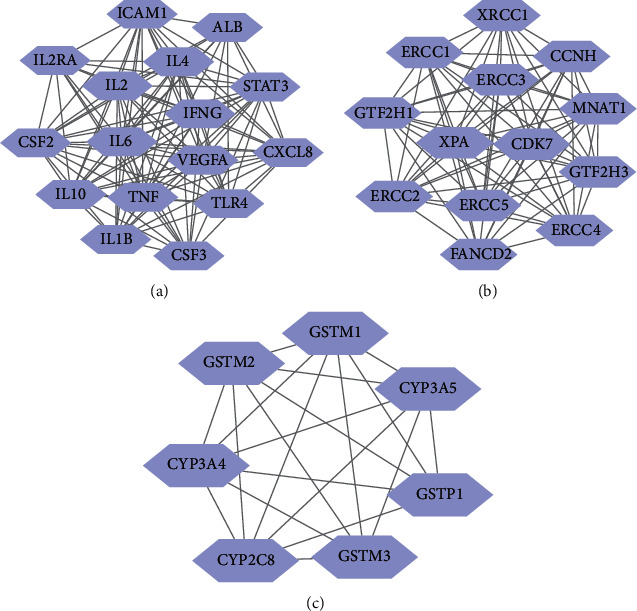
Mcode clustering of CIPN targets in the protein-protein interaction network. (a–c) represent clusters 1, 2, and 3, respectively.

Through the modular analysis of the CIPN network using the Mcode cluster, the entire network was divided into three clusters (Figure 2).

**Figure 3:**
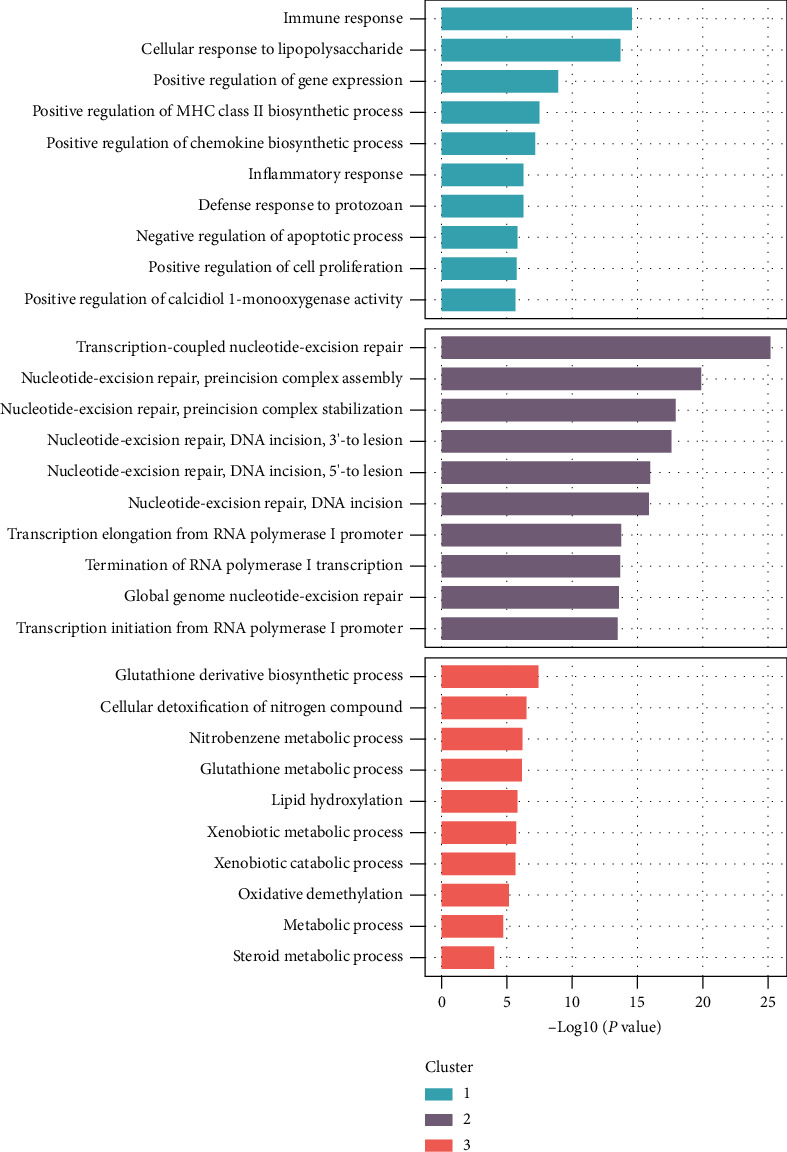
GO biological process analysis of CIPN clusters.

These clusters were then interpreted using GO biological process (GO-BP) analysis. The top 10 most significantly enriched BP terms in each group were selected for analysis. The results showed that cluster 1 included the immune response, cellular response to lipopolysaccharide, positive regulation of nitric oxide biosynthesis, positive regulation of chemokine biosynthesis, and inflammatory response. Cluster 2 included mechanisms involving transcription-coupled nucleotide-excision repair, transcription elongation from the RNA polymerase I promoter, nucleotide-excision repair, and DNA incision. Cluster 3 included processes involved in glutathione derivative biosynthesis, cellular detoxification of nitrogen compounds, glutathione metabolism, and xenobiotic metabolism (Figure 3).

### 3.3. KEGG Pathway Enrichment Analysis of CIPN Targets

**Figure 4:**
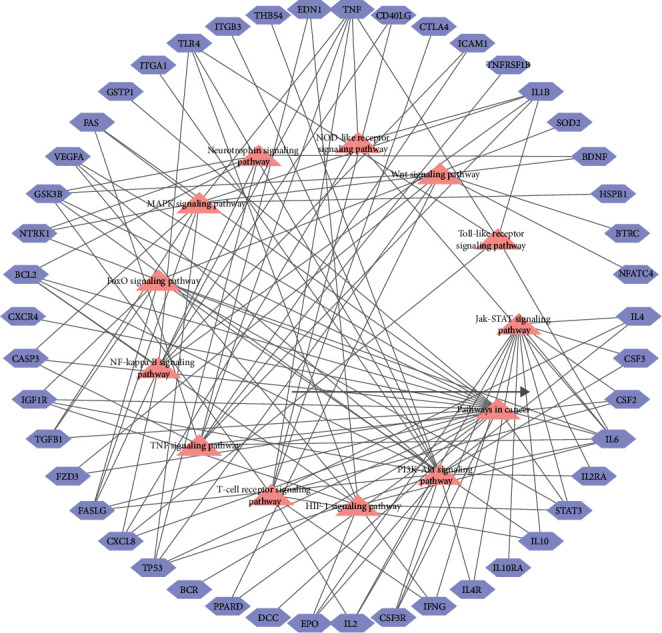
KEGG pathways of CIPN targets.

All disease genes were uploaded to DAVID, and 13 pathways were obtained, of which four pathways contained more enriched genes (i.e., 20 in pathways in cancer, 18 in the PI3K-Akt signaling pathway, 13 in the Jak-STAT signaling pathway, and 10 in the MAPK signaling pathway) (Figure 4).

### 3.4. Herbal-Compounds-Targets Network of HQGZWWD

**Figure 5:**
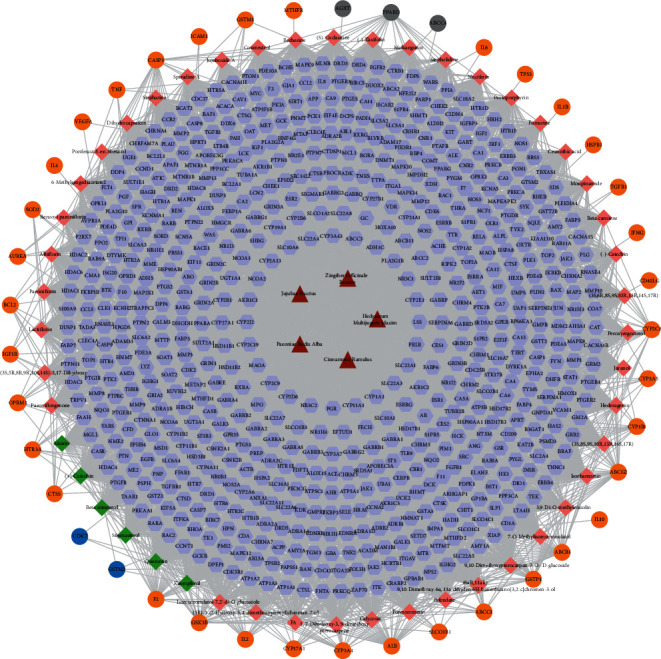
Herbs-compounds-targets network of HQGZWWD. Red triangle nodes represent herbs, diamond nodes represent compounds, green diamond nodes represent repeated compounds in herbs, peripheral nodes represent overlapping targets between HQGZWWD and CIPN, gray round nodes were not identified in the previously constructed CIPN targets network, and blue rounds represent targets derived from predictive data.

This network consisted of 728 nodes (5 herbal nodes, 48 compound nodes, and 675 compound-target nodes) and 5448 edges, of which 6 compounds (*mairin, beta-sitosterol,* (*+*)*-catechin, quercetin, stigmasterol,* and *kaempferol*) appeared in more than two herbs, and many targets (such as GSTM1, GSTM2, ACHE, and ESR2) that were targets of multiple compounds. In addition, 41 overlapping targets (peripheral nodes, such as CYP2C8, GSTM1, AGXT, GSK3B, and ABCC4) were identified after comparing herbal formula targets with CIPN targets, of which three targets (AGXT, ABCC4, and PPARD) were not identified in the previously constructed disease network, and two targets (CDK7 and GSTM2) were obtained from the predictive platform. Finally, using the DAVID platform, we found that the targets in herbal formulations were enriched in a variety of diseases such as lung cancer, type 2 diabetes, bladder cancer, chronic obstructive pulmonary disease, colorectal cancer, and chronic renal failure (Figure 5).

### 3.5. HQGZWWD-CIPN Overlapping Targets Network

**Figure 6:**
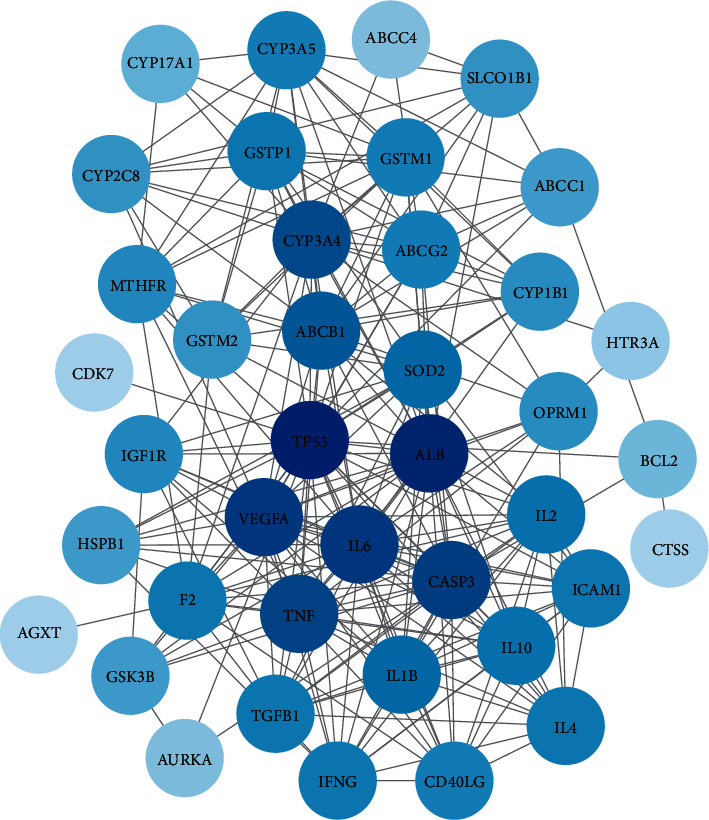
Overlapping targets network. The node having a darker color has a higher degree in the network.

The above 41 overlapping targets were inserted into the PPI network and denominated the HQGZWWD-CIPN common target network. A total of 40 nodes and 247 edges were constructed, in which 7 targets had a higher degree in the network (29 in TP53, 27 in ALB, 23 in IL6, 23 in VEGFA, 22 in CASP3, 21 in TNF, and 20 in CYP3A4). These nodes were predicted to play a core role in the treatment of CIPN with HQGZWWD (Figure 6).

### 3.6. GO Functional and KEGG Pathway Enrichment Analysis of Overlapping Targets

**Figure 7:**
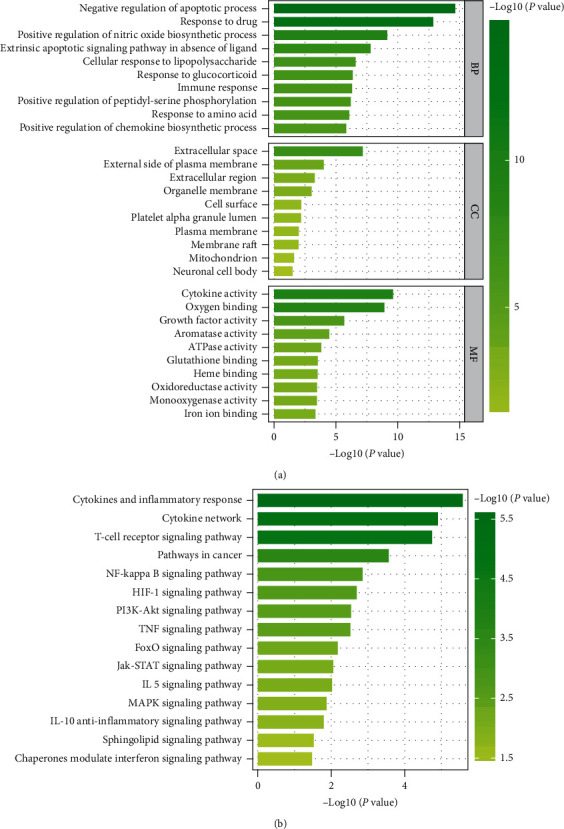
(a) GO functional analysis of overlapping targets. (b) KEGG pathway enrichment analysis of overlapping targets.

To study the biological functions and metabolic pathways of these key targets, DAVID analysis was employed to analyze overlapping targets. GO functional analysis revealed the following target-associated terms: (i) “biological processes (BP)” involving negative regulation of apoptosis mechanisms, immune responses, positive regulation of chemokine biosynthetic processes, and positive regulation of nitric oxide biosynthetic processes; (ii) “cell components (CC)” comprising the extracellular space, external side of the plasma membrane, and extracellular region; and (iii) “molecular functions (MF)” involving cytokine activity, oxygen binding, growth factor activity, and glutathione binding. KEGG pathway enrichment analysis showed that the overlapping targets were mainly involved in cytokine and inflammatory responses as well as the T-cell receptor, NF-kappa B, HIF-1, TNF, and Jak-STAT signaling pathways (Figures 7(a) and 7(b)).

## 4. Discussion

At present, the mechanisms involved in CIPN have not been fully elucidated and may include disruption of neuronal axonal transport, mitochondrial dysfunction, inflammatory stimulation, oxidative stress, nerve injury, and changes in ion channel activity. The pathogenesis of CIPN is mainly associated with injury to sensory neurons in the dorsal root ganglion caused by chemotherapeutic drugs. The incidence and severity are not only related to individual factors but also include the type of agent, cumulative dose, treatment schedule, and treatment time [30]. The classic treatment strategy is still prevention and management of symptoms [31]. Ion channel modulators, neuroprotective agents, antioxidants, tricyclic antidepressants, and antiepileptic agents are commonly used in clinical treatment [32, 33]. The only agent currently recommended for the treatment of neuralgia caused by CIPN is duloxetine [34, 35]. A “stop-and-go” strategy is generally adopted, which involves stopping treatment with the drug immediately, reducing the drug dose, or prolonging the time of chemotherapy and then resuming treatment after the symptoms are relieved [36].

Through previous animal studies, we determined that HQGZWWD could reduce the intake of platinum in the dorsal root ganglion of the oxaliplatin rat model and could promote platinum pumping, so as to reduce accumulation of platinum and prevent chronic peripheral neurotoxicity induced by exposure to oxaliplatin [37]. A meta-analysis showed that HQGZWWD could effectively prevent and reduce oxaliplatin-related peripheral neurotoxicity [38]. Therefore, we propose that HQGZWWD can effectively reduce and alleviate the incidence and severity of CIPN. Through network pharmacology, we constructed the core networks involved in CIPN and mechanisms of action of herbal formulations to explore the underlying pathogenesis of CIPN and to explore any molecular therapeutic mechanisms and/or pathways potentially associated with HQGZWWD.

First, we constructed and analyzed the core network of CIPN, in which regulatory genes including *STAT3, IL6, TNF, IL10, VEGFA, TP53, IL2, IL4*, and *CSF2* were identified. Among these was *STAT3*, which not only participates in the signal transduction pathways of many cytokines including interferon, interleukins, and growth factors but also regulates important functional activities such as cell growth, differentiation, migration, apoptosis, autophagy, immunity, and metabolism. Studies have shown that *STAT3* is not only involved in the inflammatory response but also mediates tumorigenesis and stages of carcinogenesis [39, 40]. Furthermore, *STAT3* also has an effect on peripheral nerve cell regeneration and participates in nerve repair [41]. The cytokines IL6, TNF, IL10, IL2, and IL4 are all immunomodulatory factors, among which TNF and IL-6 have been confirmed to be involved in the pathological process of peripheral nerve injury [42, 43]. The core biological processes of CIPN include the immune response, positive regulation of nitric oxide biosynthesis, inflammatory response, and positive regulation of chemokine biosynthesis. Nitric oxide exerts both anti-inflammatory and proinflammatory regulatory effects. Its proinflammatory activity is mainly manifested in the promotion of the proliferation of inflammatory cells and tissue injury. An imbalance in nitric oxide levels is the key factor causing neuropathic pain [44]. Through pathway enrichment analysis, we found that most genes we identified were enriched in inflammation-related pathways, including the Jak-STAT, NF-kappa B, MAPK, and Toll-like receptor signaling pathways. The Jak-STAT signaling pathway is widely involved in cell proliferation, differentiation, and apoptosis, which can promote the occurrence and development of inflammation and tumors. Dominguez et al. [45] found that this pathway can be activated in the spinal cord microglia of rats with peripheral nerve injury and leads to neuropathic pain. Essentially, we found that the main pathological processes of CIPN may involve the inflammatory response and nerve injury, and our results also indirectly explain the complexity of CIPN pathogenesis, and these key pathways provide a direction for the future development of new drugs.

Secondly, we explored the herbal compounds and their targets. Our results showed that HQGZWWD comprises a variety of active compounds. For example, quercetin is a flavonoid widely found in herbs, which exerts antioxidative, anti-inflammatory, antiallergic, and analgesic effects [46]. Recent studies have shown that quercetin can reduce the growth and invasion of tumor cells [47, 48]. Quercetin has anti-inflammatory activity by inhibiting the production of cyclooxygenase (COX) and lipoxygenase (LOX), which are considered to be closely related to inflammation [49, 50]. In addition, this compound shows remarkable antinociceptive and neuroprotective effects in animal models and inhibits light edema formation [51, 52]. Another molecule identified was catechin, which has anti-inflammatory and antioxidant activities and can reduce abnormal sensation after chemotherapy [53, 54]. Other active compounds identified, such as formononetin [55], isorhamnetin [56], and kaempferol [57], have been shown to be effective in inhibiting inflammation and analgesia. It is worth noting that we found that the targets of HQGZWWD can be mapped to the endocrine system and to respiratory system diseases, which are especially enriched in cancer. In recent years, TCM has attracted much attention in improving cancer and its side effects, which is consistent with our results suggesting that HQGZWWD exerts pharmacological effect on many diseases, including CIPN, through the synergistic action of many compounds and different targets. This may define this herbal formula as having multitarget and multifunction characteristics.

Finally, we targeted HQGZWWD and CIPN, as a key approach in excavating the core treatment mechanism of the TCM. We found that *TP53, ALB, IL6, VEGFA, CASP3, TNF*, and *CYP3A4* may play core regulatory roles in treatment. Among these, *TP53* is a highly tumor-related gene. Mutant *TP53* promotes the proliferation, migration, survival, and invasion of tumor cells, enhances drug resistance, and promotes the metabolism of tumor cells [58]. Both CASP3 and CASP4 belong to the caspase family, which can maintain homeostasis by regulating apoptosis and inflammation [59, 60]. VEGFA can protect neurons by promoting neovascularization and vascular permeability. Vencappa et al. [61] demonstrated that VEGFA could prevent cisplatin-induced sensory neuronal damage. In addition, inflammation-related targets such as IL6 and TNF are also core targets of CIPN. The GO function analysis of overlapping targets showed that the main biological processes of these targets were the positive regulation of nitric oxide biosynthesis, immune responses, and responses to glucocorticoids, which are similar to CIPN. While KEGG pathway enrichment analysis showed that the targets that were mainly enriched included cytokines and inflammatory response, elements of the cytokine network, and the T-cell receptor, NF-kappa B, HIF-1, and PI3K-Akt signaling pathways, which were mostly associated with the inflammatory response. For example, the HIF-1 signaling pathway participates in many biological processes such as hypoxia adaptation, inflammatory development, and tumor growth and plays a key role in inflammation and mediates tumorigenesis [62, 63]. We found that these pathways intersect with the disease pathway of CIPN, indicating that HQGZWWD has a certain pertinence in the treatment of CIPN.

Overall, our results show that HQGZWWD plays a therapeutic role in CIPN by regulating the inflammatory response and repairing nerve injury, thus providing support for the reliable efficacy of this herbal formula. In addition, in the overlapping targets network, we identified two potential targets (CDK7 and GSTM2) from the prediction platform, which have not been verified by experiments, and represent a clear direction for future research as novel targets for the treatment of CIPN.

Our innovation in this study lies in our integration of multiple TCM databases, which may compensate for the deficiency of incomplete data. Further, our supplementary analysis of the targets for CIPN treatment through a prediction model provides new information for the future study of pathological processes and therapeutic targets. Finally, this study fully illustrates that TCM possesses the characteristic of an active multicompound, multitarget, and multipathway formula, which is of great significance in the study of the curative effect of herbal formulations. However, there are some issues that cannot be overlooked; although the selection of effective compounds is well founded (mainly recommended by databases), they are mostly reported in a single plant, which does not mean that they are still present and play a therapeutic role in the traditional preparation. As mentioned above, we found only about 30% of the effective compounds in a mass spectrometric analysis of HQGZWWD. Therefore, more mass spectrometric analysis needs to be carried out to determine the effective compounds of traditional preparations.

## 5. Conclusion

Through network pharmacology, we found that HQGZWWD has a significant advantage in the treatment of CIPN. At the same time, the underlying molecular biological mechanisms have been revealed by analyzing the potential core targets, biological functions, and signal pathways involved. Our study provides a theoretical basis for the clinical application of HQGZWWD for the treatment of CIPN.

## Data Availability

The data used to support the findings of this study are available from the corresponding author upon request.

## Supplementary Materials

Supplementary Materials Supplementary 1. Table S1: compounds identified in the HQGZWWD formula. Supplementary 2. Table S2: effective compounds identified in the HQGZWWD formula. Supplementary 3. Table S3: targets of the HQGZWWD formula. Supplementary 4. Table S4: targets of CIPN.
